# A List of Cases Discharged from the Surgical Wards of the Pennsylvania Hospital, during the Month of May, 1843

**Published:** 1843-06-24

**Authors:** 

**Affiliations:** Attending Surgeon


### A LIST OF CASES

### DISCHARGED FROM THE SURGICAL WARDS OF THE PENNSYLVANIA HOSPITAL,

*During the month of May*, 1843.

DR. NORRIS, Attending Surgeon.

[Reported by ELLERSLIE WALLACE, M. D., Resident Surgeon.]

1 case of fracture of the femur, occurring in a lad æt. 17 years. He had anchylosis of the knee joint, of some years standing, with considerable flexion of the leg. The seat of fracture was about four inches above the knee joint. He was treated by a roller from the toes to the groin, and a pair of splints were applied to the thigh, keeping the fragments in good apposition. The patient was placed on his back and the limb supported by pillows.

In thirty-five days from the receipt of the injury he was allowed to move about with crutches, and in forty-nine days he was discharged, a complete cure having been effected.

1 case of fracture of the outer condyle of the humerus, in a boy aged four years. Treated by a roller from the ends of the fingers to the axilla, and an angular splint on the inner side of the upper extremity. Passive motion was commenced in ten days, and the splint was removed in twenty-one days. After being under treatment twenty-nine days, he was discharged cured.

1 case of old standing caries of the femur. Discharged at her own request.

1 case of a severe incised wound immediately above the patella, caused by the glancing of an axe. The man was admitted 36 hours after the accident, having been brought twenty-five miles in a wagon. The wound had been covered with a handful of salt, immediately after the injury, and at the time of his entrance into the hospital, was found to be in a high state of inflammation. The limb was elevated and poultices were applied until the granulations assumed a healthy appearance; it was afterwards treated as an ordinary ulcer. Under treatment thirty-eight days. Discharged cured.

3 cases of injury from blasting rocks. In two of the patients the face was the principal seat of the injury, resulting in one case in total loss of vision; the other recovered with partial sight of one eye. In the third case, the leg was severely contused, and somewhat lacerated. At one of the contusions a deep slough formed, which separated on the application of the fermenting poultice, leaving a healthy granulating surface beneath. Treated afterwards as a common ulcer. At his own request, he was discharged in thirty-nine days, nearly well. (The fermenting poultice used in the hospital is made of indian meal, mixed with porter to a proper consistence.)

1 case of contraction of the biceps flexor cubiti, depending on a slight inflammation of the elbow joint. Treated by a soap plaster over the joint, and a splint applied to the front of the upper extremity, by which the contraction was overcome, and in eight days he was discharged, cured.

10 cases of contusion. Several of which were slight, and were discharged cured in a few days. One case in which the knee was the seat of injury, was followed in a few days by a severe attack of acute rheumatism; removed, while under treatment, at his own request. One of the cases was caused by a severe blow on the back, in the concavity between the spine and the os ilium of the right side. When the man was admitted, he could not bear even a slight pressure on the injured part, and the least movement of the pelvis or the thigh caused him exquisite pain. There was, also, numbness to a considerable extent along the line of the anterior crural nerve of the right side, and the motions of the right lower extremity were materially impaired. An enema was ordered to open his bowels, and his urine passed during its operation. Cut cups were applied over and around the contused spot, and he was kept at perfect rest. After the lapse of two days, as some numbness still remained, he was again cupped, and the cups were followed by the application of a large plaster of burgundy pitch. He was freely purged, and was kept on a low diet. In nine days, he was discharged cured.

2 cases of lacerated wounds of small extent.

3 cases of abscesses, of small size.

2 cases of ulcer. One on the forearm, the result of a burn. Besides the local application to the ulcer, the limb was secured by a roller from the ends of the fingers, and the arm was supported in a sling. In the other case the leg was the part affected, and it was treated in a fracture box. The plan of perfect rest, in the treatment of ulcers, is enforced in the hospital, and with the most gratifying results.

1 case of enlargement of the glands of the neck. The patient was a young man, of a strumous diathesis. He was treated by the iodide of potassium internally, with the external use of the ung. iod. comp., and the part was kept covered by a soap plaster. In fifty-eight days he was discharged, cured, his general health being much improved.

1 case of hydrocele. Treated by the injection of equal parts of port wine and water, after the fluid in the cavity had been removed by the trocar. The affection was of long standing, and the fluid amounted to about f℥ix. He was discharged, cured, in twenty-six days.

1 case of lupus, in a woman aged 42, in which the tip, and a part of the alæ of the nose had been destroyed previous to her admission. The inflammation had extended to some distance around the part affected. The treatment consisted chiefly in the local application of a poultice of powdered slippery elm at night, and of a pledget of lint wet with lime water during the day. A solution of sulphate of copper ℈j. to water f℥j. was occasionally applied by a camel's hair pencil. The iodide of potassium was administered internally, in doses of gr. x. in syrup sarsaparilla c. f℥ss. three times a day. The progress of the disease was arrested after a few days, and after being under treatment forty-four days, she was discharged, cicatrization being perfect.

From the venereal ward *three* cases were discharged. One was a case of a succession of very firm strictures of the urethra, of five years standing. They were treated by dilatation; during the treatment an abscess formed in the perineum, requiring the use of the bougie to be suspended for some time. Upon the healing of the abscess, the treatment was recommenced, and an instrument of good size could be passed to within about an inch of the neck of the bladder, where a very tight stricture was found, which was gradually yielding, when, at his own request, he was discharged.

Besides the above, the following cases were treated, and discharged on the day of their admission, at their own request.

3 cases of incised wounds of the hand.

1 case of paronychia. Treated by a free incision and the application of a poultice.

1 case of fracture of the radius. Dressed with a splint, on the front and back of the fore arm and hand.

